# Utilization of Organic Solvents for the Recycling of Waste Wooden Railroad Ties

**DOI:** 10.3390/molecules31030406

**Published:** 2026-01-24

**Authors:** Željka M. Nikolić, Miloš S. Tošić, Jelena M. Radivojević, Mihajlo Gigov, Milica P. Marčeta Kaninski, Vladimir M. Nikolić, Dragana Z. Živojinović

**Affiliations:** 1 Institute of General and Physical Chemistry, Studentskitrg 12/V, 11000 Belgrade, Serbia; jradivojevic@iofh.bg.ac.rs (J.M.R.); mgigov@iofh.bg.ac.rs (M.G.); milica@iofh.bg.ac.rs (M.P.M.K.); vnikolic@iofh.bg.ac.rs (V.M.N.); 2 Vinča Institute of Nuclear Sciences, Mike Petrovića Alasa 12-14, 11000 Belgrade, Serbia; milos.tosic@vin.bg.ac.rs; 3 Faculty of Technology and Metallurgy, University of Belgrade Karnegijeva 4, 11000 Belgrade, Serbia; gaga@tmf.bg.ac.rs

**Keywords:** railroad ties, wood waste, solvent extraction, waste management, polycyclic aromatic hydrocarbons

## Abstract

Wooden waste railroad ties preserved with coal tar creosote oil represent a specific source of polluting substances. The aim of this study was to investigate and compare extraction capacity due to solvent extraction of fifteen frequently used organic solvents for the purpose of decontamination treatment of waste wooden railroad ties, while recovering wood for reuse. Pure organic solvents, ethanol 96%, propan-2-ol, deionized water, dichloromethane, acetone, *n*-hexane, mixture *n*-hexane/acetone (V/V = 1/1), cyclohexane, methanol, N,N-dimethyl formamide, toluene, ethyl acetate, acetonitrile, amyl acetate, medical gasoline, *n*-pentane and *n*-butyl acetate were for leaching pollutants from waste railroad ties. The highest extraction capacity was achieved using dichloromethane, where 7.50 to 7.89 wt.% of total sixteen polycyclic aromatic hydrocarbons were extracted from waste railroad tie chips. The most promising solvents for the treatment exhibited extraction efficiency which decreases in a series dichloromethane > *n*-hexane/acetone > acetone > methanol > ethanol 96% > propan-2-ol > cyclohexane > toluene > *n*-hexane. Solvent extraction represents a novel approach for treatment of wooden waste railroad ties. The experiments are based on the search for a management process for the treatment of wood waste railroad ties that is simple, low energy consumption, efficient and could potentially be applied for large scale.

## 1. Introduction

Wood is a completely natural, biological, widely applied construction material and renewable resource in human society, yet high affinity for water makes it susceptible to attack by biological agents, such as bacteria, insects and fungi causing degradation. Fungal decay is the most predominant failure mode of wooden products. Chemical agents for wood protection are systematized as oil-based and water-based wood preservatives [1,2]. Coal tar creosote oils are oil-based wood preservatives and have been used in industry from 1838 with John Bethell’s full cell process of wood impregnation [3]. Primarily, creosote oils are used for wooden railroad ties preservation, wooden fences, wooden telephone poles preservation, etc. In terms of chemical composition, creosote oils are a complex mixture of chemical compounds that can contain up to 85% polycyclic aromatic hydrocarbons (PAHs), 10% phenolic compounds and 5% heterocyclic compounds containing nitrogen, sulphur or oxygen [4].

Polycyclic aromatic hydrocarbons are major components in creosote oils. There are sixteen PAHs that are priority pollutants, established by the U.S. Environmental Protection Agency (EPA) [5]. Laboratory analyses, supervised by the Swedish Environmental Protection Agency, determined that sixteen U.S. EPA PAHs can be found in creosote in a maximum 15% of mass fraction [6]. The European Union (EU) in 2003 prohibited the sale of creosote-treated wood to the general public [7]. The use of creosote in the United States is restricted to outdoor applications, while indoor use is prohibited [8]. PAHs are considered as pollutants and toxic substances and can be found in water [9], air [10], soil [11], sediments [12], sludge [13] and food [14,15]. PAHs are mainly formed through the following four different mechanisms: combustion (cigarette smoking, exhaust gases) [16,17,18], pyrolysis [19,20], pyrosynthesis of organic material [21,22] and diagenesis processes [23,24]. The toxicity of certain PAHs includes carcinogenic, mutagenic, immunological and reproductive effects [25,26]. In the environment, they are the most persistent in soil and sediments [27,28]. Due to high wt% of PAHs in coal tar creosote, it is classified as Group 2A (probably carcinogenic to humans) by the International Agency for Research on Cancer [29].

Wooden railroad ties impregnated with creosote are often viewed as a material capable of emitting relatively small quantities of potentially harmful substances into the environment (air, soil, water) while they are located on railway embankments. The emitted pollutants showed higher mobility in sediments than in soil, resulting in higher concentrations in soil [30,31]. The PAHs from creosote in wooden railroad ties reach the environment through the processes of leaching, dripping, diffusion, abrasion and evaporation [32]. During service life, an average of 25–30 years, each wooden tie emits about 5 kg of creosote into the environment. Priority pollutants, 16 PAHs, account for between 20% and 40% of the total weight of a typical creosote [33]. The release of creosote occurs non-linearly; the more volatile PAHs (naphthalene, acenaphthene, fluorene and anthracene) are emitted first. After about 1 year, less volatile PAHs are secreted or released through powdery substances [32,34].

When it comes to replacing railroad ties, it becomes a waste, under the U.S. EPA Non-Hazardous Secondary Materials (NHSMs) rule from 2011, including updates from 2013, 2016 and 2018 and carries a certain risk to the environment [35]. Waste wood material is considered valuable because it can be reused, recycled or used as an energy resource [36]. In Europe, 33,700 m^3^ of wood products became waste in 2010. Amongst the specified quantity, 46% of wood waste is recycled and 51% is incinerated [37]. In France, approximately 50,000 t of used wooden ties were incinerated in 2012 in order to save energy. Wooden waste railroad ties (WRTs) have enhanced heat value because of creosote impregnation. Kim et al. [38] have shown that thermal desorption of creosote at temperature of 280 °C for 10–30 min shows recovery level of creosote comparable to that at 300 °C, with 20–25% weight loss and energy yield 77–83%. Thermal treatment of WRTs conducted at temperatures between 250 and 350 °C, where the amounts of creosote, mostly consisting of polycyclic aromatic hydrocarbons (PAHs), were recovered from 47 to 79% of total creosote present in the used ties, as indicated by [39].

Thermo-chemical treatments such as pyrolysis, torrefaction or gasification are suitable methods for transformation of used WRTs to obtain solid fuel or biochar. Torrefaction up to 250 °C gives high efficiency of impregnation removal, while pyrolysis up to 400 °C produces waste that can be considered safe. The obtained chars were submitted to Soxhlet method solvent extraction to monitor the extraction process [40]. In the study by Gonzalez et al. [41] researchers investigated the pyrolysis of creosote-treated railroad ties to recover creosote and to develop biochar. Under pyrolysis at 700 °C, 80.7% of the Ʃ16 U. S. EPA PAHs were recovered in the condensates. Fast pyrolysis of creosote-treated wood ties in a fluidized bed reactor produces the following three fractions: oil, gas and char, according to Jung et al. [42]. Mun et al. [43] have conducted air gasification of WRTs treated with creosote to produce a clean and high caloric gas with low tar content.

WRTs disposed of in landfills have negative environmental impacts, namely leaching of hazardous pollutants and emission of methane [44,45]. Survey results indicate that approximately 16.5 million ties in 2017 were covered by the following ten types of management: reuse by same railroads (1.0%), reuse by other railroads (0.1%), reuse commercial landscape (12.5%), reuse agriculture (4.1%), reuse residential landscape (10.4%), other (0.0%), incineration (0.0%), recycle combustion (for energy) (65.7%), recycle gasify (for energy) (0.2%) and landfill (6.0%) [46].

However, use in households and institutions, parks or gardens leads to higher human exposure to compounds present in impregnation agents. Ikarashi et al. [47] showed that wooden railroad ties, brand-new or secondhand, contain large amounts of benzo[a]pyrene (58–749 μg/g) and benz[a]anthracene (250–1282 μg/g). In the work of Mateus et al. [48], the volatile fraction of waste creosote-treated railroad ties sample was extracted by purge-and-trap at ambient temperature. Qualitative analysis showed that the compounds present as volatile components are phenol and benzene derivatives, PAHs and N-, S-, O-heterocyclic compounds and their derivatives. A study was conducted to measure the concentration of sixteen priority PAHs and water-extractable phenols in wooden ties put into operation between 1936 and 1978 in France [49]. The results showed that the creosote content in all samples was well above 1000 mg/kg, even after 76 years. These results showed that wooden ties should be treated as hazardous waste.

WRT waste management contains reuse, recycling and disposal with limitations because of hazardous substances. It can be reused for landscaping, fencing, outdoor steps or rustic decoration. It is not suitable for indoor use, mulch, compost, garden beds or burning in household stoves or fireplaces. Recycling is carried out at specialized recycling centers which can process them as biomass fuel. Approximately 65% of the total creosote remains in railroad wood ties after 35 years of service [38]. Reuse of WRTs, such as reinforcing agents or fillers in cementitious composites, has advantages and numerous positive effects such as low density, low price, natural resistance to abrasion, possible use in a higher percentage and low energy consumption. Recycling used wood products is a way to reduce the amount of waste and the demand for a fresh natural resource [50,51].

Solvent extraction is a purification method including different solvents used to extract or remove harmful compounds from contaminated materials. When matrix material is in solid state, the process is called leaching [52,53]. Solvent extraction can be performed with pure solvents, primarily organic solvents, or with its mixtures. Solvents are chosen in correlation with the contaminant’s physical and chemical properties. Remediation of soil contaminated with PCP could be performed using an ethanol–water mixture as a solvent [54]; if contaminants are polychlorinated dibenzo-p-dioxins and PAHs, an alkane-alcohol mixture could be used [55]. Extraction of chlorinated compounds and hydrocarbons from soil can be achieved using a solvent mixture of ethyl acetate–acetone–water or using the hexane–acetone solvent system [56,57]. Solvent extraction technique is also applied during sample preparation for chromatographic separations. Determination of PAHs concentration in surface soils includes sample preparation with extraction techniques such as ultrasonication with acetone–methanol dissolution [58], ultrasonication for 30 min with the mixture of dichloromethane/acetone [59], hexane, acetone/hexane and chloroform/methanol [60]. Nishi et al. investigated extraction of PAHs from creosote-treated wood with organic solvents to find if acetone, acetone/hexane and hexane could be used as a solvent alternative to dichloromethane in sample preparation [61].

The technology of purifying contaminated material by solvent extraction has not yet been applied to WRTs. In this study, we have investigated several approaches based on solvent extraction methods for treatment of WRTs. The goal was to reduce the concentration of pollutants in the WRT chips to the point where the wooden WRTs ceased to be harmful to the environment. Pure organic solvents were used as a liquid phase for leaching tests and Soxhlet extraction. Solvent extraction method with soaking, without shaking, at room temperature does not require any additional energy consumption. except energy for preparation WRTs for extraction (grinding in the mill). The extraction process requires time because it is controlled by the diffusion mass transfer phenomenon [47,58]. Products at the end of treatment would be refined or partially decontaminated wooden chips intended for further use and extract that can be separated by fraction distillation into two fractions: creosote and solvent. This study shows potential for savings on landfilling space, reduced possibility of contamination by leaching and reduced chemical transformations of pollutants caused by high temperature.

## 2. Results and Discussion

**Figure 1:**
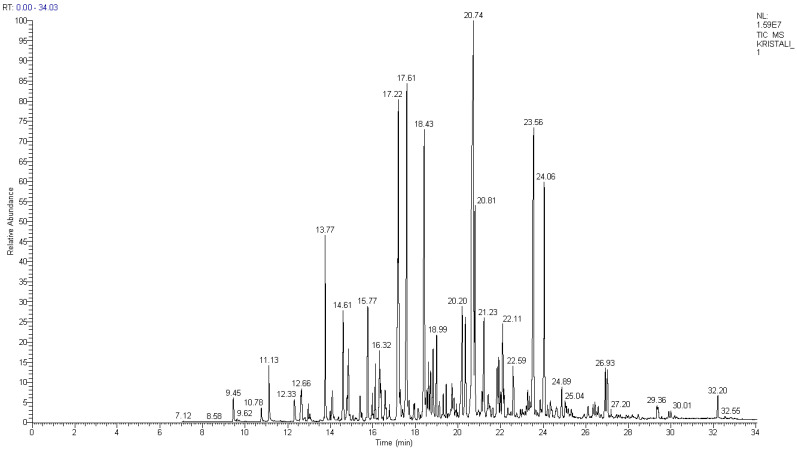
GC-MS TIC full-scan chromatogram of WRT extract in dichloromethane.

Creosote oil is a complex mixture of chemical compounds that can contain up to 85% polycyclic aromatic hydrocarbons (PAHs), 10% phenolic compounds and 5% heterocyclic compounds containing nitrogen, sulfur or oxygen. Qualitative analysis of WRT test sample dichloromethane extract was performed by GC-MS technique and NIST compounds library was employed to identify organic polluting substances. GC-MS TIC full-scan chromatogram of WRT extract in dichloromethane is presented in Figure 1. List of identified analytes is shown in Table S1 in the Supplementary Material.

Some of the analyte signals with the highest intensity were derived from 16 U.S. EPA PAHs. Identified and quantified PAHs along with their retention time exhibited in Figure 1 are naphthalene (12.96 min), acenaphthene (17.22 min), fluorene (18.43 min), phenanthrene (20.74 min), anthracene (20.81 min), fluoranthene (23.56 min), pyrene (24.06 min), benz[a]anthracene (26.93 min), chrysene (27.01 min), benzo[b]fluoranthene, benzo[k]fluoranthene (29.36 min) and benzo[a]pyrene (30.01 min). Among 16 U.S. EPA PAHs acenaphthylene, benzo[ghi]perylene, dibenz[a,h]anthracene and indeno[1,2,3-cd]pyrene have not been identified. Group of seven PAHs (benz[a]anthracene, benzo[a]pyrene, benzo[b]fluoranthene, benzo[k]fluoranthene, chrysene, dibenz[a,h]anthracene and indeno[1,2,3-c,d]pyrene) is classified as potentially carcinogenic to humans according to IARC. Five of them are present in dichloromethane extract of the WRT test sample.

### 2.1. Soxhlet Extraction of WRT Chips

One group of experiments represents solid–liquid extraction known as Soxhlet extraction technique performed on wooden WRT chips. The following solvents were employed for this purpose։ ethanol 96%, propan-2-ol, deionized water, dichloromethane and acetone. During experiments, test samples were placed in an extraction chamber, and solvent was heated till its boiling point in eight cycles. Results are presented in Table 1.

Solvent mass fraction retained on the test sample, Δm/m_0_, represents values in range from 0.41 to 0.47 and the highest value stands for dichloromethane. The average calculated value of solvent mass fraction retained on the test sample is 0.46. The lowest value 0.41 was calculated in case of deionized water. A certain amount of solvent retained on the walls of the extraction chamber and additional amount have been absorbed by cellulose extraction thimbles which impacts the overall results in retained solvent mass fraction. Solvent fraction retained on the test sample was determined to consider whether a solvent has a pronounced power of wetting and binding to the wood matrix. Derived data indicated that there were no specific wetting agents among tested solvents.

### 2.2. Solid–Liquid Extraction (Leaching) Through Soaking, Without Shaking

One set of experiments represents solid–liquid extraction whereas the 1 g of sample was soaked in different organic solvents for period of 24 h at room temperature, 25 °C, presented in Table 2. Solvents used for extractions were ethanol (96%), propan-2-ol, deionized water, dichloromethane, acetone, *n*-hexane, mixture *n*-hexane/acetone (V/V = 1/1), cyclohexane, methanol, N,N-dimethyl formamide, toluene, ethyl acetate, acetonitrile, amyl acetate, medical gasoline, *n*-pentane and *n*-butyl acetate.

**Figure 2:**
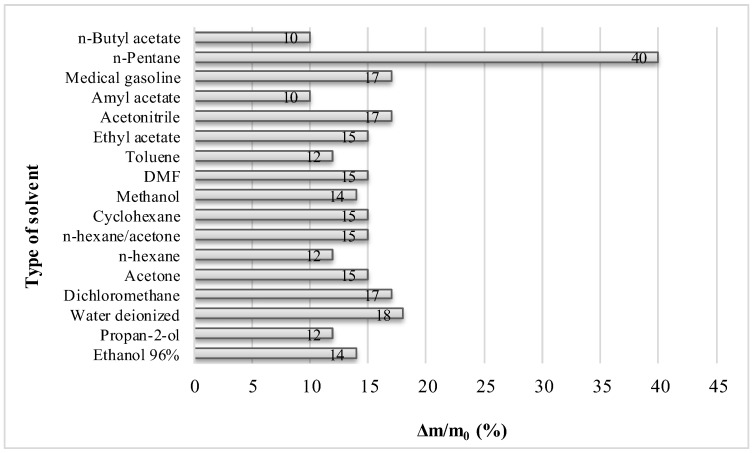
Solvent mass fraction retained on test sample, Δm/m_0_, in relation to type of solvent.

Solvent mass fraction retained on the test sample, Δm/m_0_, represents what part of the starting mass of solvent remained on the sample. Its values are in range from 0.10 to 0.18 and the most often calculated value is 0.15 (for solvents acetone, *n*-hexane/acetone, cyclohexane, DMF and ethyl acetate). The highest values stand for deionized water and dichloromethane, value 0.40, which corresponds to *n*-pentane, is probably due to high vapor pressure and low boiling point of *n*-pentane and is not essentially the measure of solvent retention, as it can be seen in Figure 2.

The average calculated value of solvent mass fraction retained on the test sample is 0.14, except in case of *n*-pentane. The lowest values were calculated 0.10 in case of esters *n*-butyl acetate and amyl acetate. Solvent fraction retained on the test sample was determined to consider whether a solvent has a pronounced power of wetting and binding to the wood matrix. Derived data indicated that none of the tested solvents exhibited a specific affinity for the wooden matrix.

**Figure 3:**
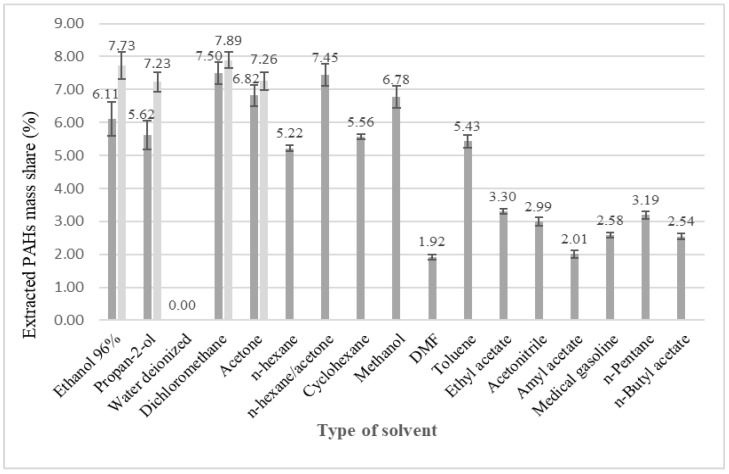
Mean extracted mass share (%) of 16 U.S. EPA priority PAHs in test samples using different organic solvents. Error bars represent the standard deviation (SD) of three independent measurements (n = 2). Light gray bars indicate Soxhlet extraction, while dark gray bars indicate soaking extraction.

Extraction of contaminants took place in 24 h; the extract was filtered through cellulose paper by means of gravity. An aliquot of 1 cm^3^ was taken for GC-FID analysis of sixteen U.S. EPA PAHs. Separation and quantification of analytes in obtained extracts was performed via GC-FID analytical technique. The moisture content in wooden WRT chips was determined and 99.98% was dry matter. Sixteen U.S. EPA PAHs were determined as pollutants in WRT chips through extraction processes in different organic solvents. PAH recovery from WRT chips is presented in Figure 3. as extracted mass share (calculated on dry matter) of sum of sixteen US EPA PAHs in test samples for soaking investigation (dark gray) and Soxhlet (light gray) extraction.

The extracted mass share of sum of sixteen U.S. EPA PAHs (EMS) was calculated using the following Equation (1):

(1) $$EMS\;\left(\%\right)=\frac{\Sigma PAHs\;(\frac{\mu g}{{cm}^{3}})\times D_{f}\times V({cm}^{3})}{m_{s}(g)\times 10,000}$$

where ΣPAHS is total concentration of sixteen US EPA PAHs, D_f_ is dilution factor, V is extract volume and m_s_ is mass of test sample.

The obtained results were normalized considering peak area and weight soil because the amount of sample weight in Soxhlet extraction and soaking extraction were different for 4.00 g and 1.00 g, respectively.

**Figure 4:**
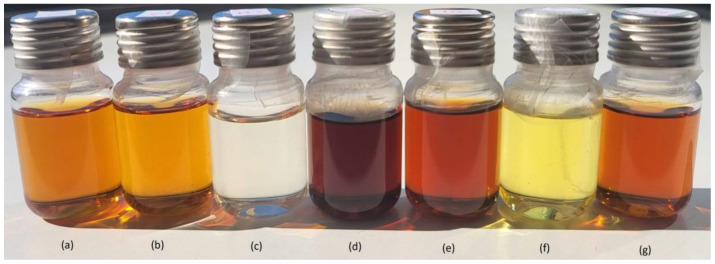
Color of the extracts after 24 h through soaking, without shaking: (**a**) ethanol 96%; (**b**) propan-2-ol; (**c**) water deionized; (**d**) dichloromethane; (**e**) acetone; (**f**) *n*-hexane and (**g**) *n*-hexane/acetone.

The extraction process could be observed through the extract color change, from transparent solvent to yellow, amber to dark brown. Color change in extract could be a variable visible to the naked eye useful for monitoring the progress of the process, as it is presented in Figure 4.

Solvents like DMF, ethyl acetate, acetonitrile, amyl acetate, medical gasoline, *n*-pentane and *n*-butyl acetate needed 12–18 h to significantly change in color that represents the extraction progress. Due to the differences in extraction rate and solvent capacity, 24 h is a time span optimal for the extraction and comparison.

Extracted mass share of sixteen U.S. EPA PAHs in test samples of WRT chips indicates that measured recovery of PAHs covers the range from <0.002 to 7.89%. The lowest measured value < 0.002% stands for deionized water as extract for both sets of experiments, soaking and Soxhlet extraction that is below the limit of detection. The highest measured value 7.89% in both sets of experiments corresponds to dichloromethane Soxhlet extract. Except deionized water, the rest of four Soxhlet extracts represent recovery over 7%, in a series dichloromethane > ethanol 96% > acetone > propan-2-ol. Measured Soxhlet recovery of PAHs in different solvents suggests each among them could be employed for the extraction process on a large scale. There would be expected small differences in the treatment process similar to Soxhlet extraction for aforementioned solvents. Extract composition shows the uniform PAHs profile (%) for soaking and Soxhlet extraction. Solvents do not show selectivity towards a specific PAH compound (data not presented).

**Figure 5:**
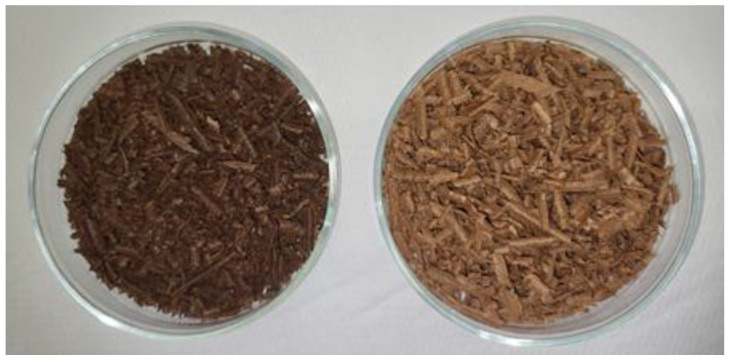
WRT chips before (**left**) and after (**right**) Soxhlet extraction with dichloromethane.

Alongside the highest extraction capacity, dichloromethane solvent through Soxhlet extraction has discolored the WRT sample and completely removed odors that originate from creosote oil. In addition to sixteen U.S. EPA PAHs, WRT chips contain other diverse organic pollutants. After Soxhlet extraction with dichloromethane and drying, wooden chips are odorless, natural wood color and loose, as it is presented in Figure 5.

Soxhlet extraction with dichloromethane was repeated with the same, previously treated chips and extract was analyzed quantitatively for sixteen U.S. EPA PAHs by GC-FID technique and qualitatively by GC-MS technique. There was no detectable signal of any organic polluting substances.

The list of qualitative extract composition is given in Table S1 in the Supplementary Material. Qualitative analysis was performed by GC-MS technique and NIST compounds library was employed to identify organic polluting substances. The organic solvents mentioned above show effectiveness not only in removing sixteen US EPA PAHs from WRT materials, but they also isolate the other organic pollutants that are present in wood chips as well. The removal efficiency of additional organic pollutants is proportional to that of polycyclic aromatic hydrocarbons. Soaking WRT chips extracts, except deionized water, maintains a wide variety in recovery of PAHs, from 1.94 to 7.50%. The highest measured value 7.50% indicates that dichloromethane maintains the highest extraction capacity among tested solvents. Second place extraction capacity 7.45% stands for *n*-hexane/acetone (V/V = 1/1) mixture. Third place extraction capacity 6.82% and 6.78% share acetone and methanol, respectively. Extraction efficiency of solvents decreases in a series dichloromethane > *n*-hexane/acetone > acetone > methanol > ethanol 96% > propan-2-ol > cyclohexane > toluene > *n*-hexane. Quantitative determination of PAHs using GC-FID technique was performed from the crude extract, so there was no need to change the reagents or clean up procedures, which means that there was no loss of analytes. The specified solvents showed the promising extraction capacity in one-stage batch for treatment WRT chips. Determined recovery of sixteen U.S. EPA PAHs in *n*-hexane extract was 5.22%. Total recovery in each individual solvent is presented in Table S2 in the Supplementary Material. Aforementioned solvents could be employed in order to accomplish leaching treatment of WRT chips at room temperature with the decrease in pollutants concentration. Additional solvent systems with Soxhlet extraction that should be performed are *n*-hexane/acetone (V/V = 1/1) mixture and methanol. The lowest recovery of PAHs 1.94%, except deionized water, was measured in DMF extract. The rest of tested solvents, ethyl acetate, acetonitrile, amyl acetate, medical gasoline, *n*-pentane and *n*-butyl acetate have measured recovery of PAHs below 3.50%, which is less than a half of the highest measured values. Deionized water came out with expected low values for PAHs extraction, due to high hydrophobicity of PAHs. Previously mentioned solvents should not be an option for treatment of WRT chips in order to remove organic pollutants.

### 2.3. Solid–Liquid Extraction (Leaching) with Shaking

The third set of experiments was based on searching for another way for treatment of WRT chips. The procedure is simple, with low energy consumption, effective and is performed at room temperature, 25 °C. Shaking will speed up the solid–liquid extraction so that it will help diffusion of pollutants into the solvent bulk. The extraction was conducted in several stages to ensure the complete removal of pollutants from the WRT chips. Extractions were performed as multistage batch solvent extraction technique with addition of fresh solvent to filtration cake in each stage, after decanting extract from previous step. Successive extractions were conducted in five stages at room temperature, 25 °C. Successive extractions were achieved till there were no PAHs detected. Total extracted mass share of sixteen U.S. EPA PAHs in test samples is presented in Table 3, along with extracted mass share of sixteen U.S. EPA PAHs in every individual stage and average extracted mass share for each extraction time.

There were five consecutive stages in dichloromethane solvent extraction of each WRT test sample. Extraction time and distinct solvent volume was associated with each series from C1 to C9. An aliquot of 1.00 cm^3^ after gravity filtration was taken for GC-FID quantitative analysis of sixteen U.S. EPA PAHs. Extracted mass share of sixteen U.S. EPA PAHs in test samples of WRT chips indicates that measured extracted mass share covers the range from 6.36 to 9.47%. An average extracted mass share for each extraction time was 7.60% in 2.00 h, 7.74% in 1.00 h and 7.50% in 0.25 h. In each set of experiments in the same extraction time, there were three distinguished sample-to-solvent ratios (5 g/100 cm^3^, 5 g/200 cm^3^ and 5 g/300 cm^3^). Measured total extracted mass share of sixteen U.S. EPA PAHs pointed out that there was no observed variation in extraction recovery due to sample-to-solvent ratio. Existing difference in extraction recovery is a consequence of the inhomogeneous distribution of organic pollutants in WRT test samples. WRT chips were well-mixed to obtain a homogenous sample in relation to the matrix and contaminants, but distribution of organic pollutants is a result of the creosote impregnation process. Measured total extracted mass share of sixteen U.S. EPA PAHs is in positive correlation with the results from 24 h extracted mass share dichloromethane leaching test (7.50%) and dichloromethane Soxhlet extraction test (7.89%). For all test samples there was a need for five extraction stages to obtain extract with no detectable leached pollutants. Results indicate that the average mass shares of sixteen U.S. EPA PAHs in test samples of WRT chips were in range from 7.50 to 7.89%.

**Figure 6:**
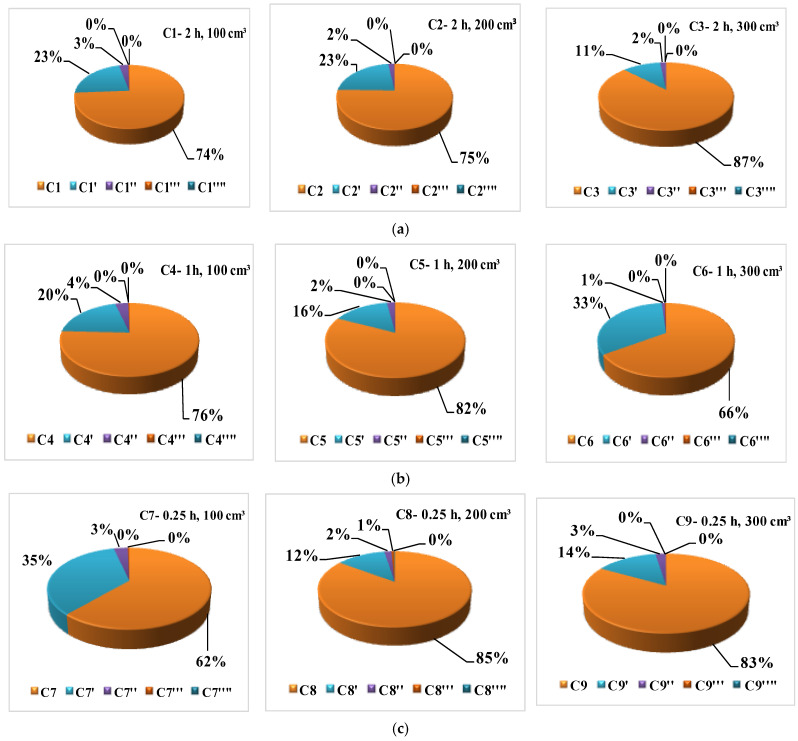
Percentage distribution of the total content of PAHs by extraction stages in solid–liquid multistage cross-current batch extraction with shaking for (**a**) 2.00 h, (**b**) 1.00 h and (**c**) 0.25 h extraction time.

Dichloromethane multistage batch leaching tests at room temperature with shaking were performed in five consecutive stages for each of WRT test samples. There were almost no detectable leached pollutants in extracts after the fourth step. Percentage distribution of the total content of sixteen U.S. EPA PAHs by five consecutive stages (marked as C, C′, C″, C‴ and C′′′′) in each series C1–C9 is presented in Figure 6a–c.

The vast majority of total PAHs present in WRT test samples was leached in the first stage. For the same extraction time of 2 h in series C1, C2 and C3, percentage distribution of the total content of PAHs was in range from 74% to 87% in the first stage. For the same extraction time of 1.00 h in series C4, C5 and C6, percentage distribution of the total content of PAHs was in range from 66% to 82% in the first stage (C) and/or the same extraction time of 2.00 h in series C7, C8 and C9, percentage distribution of the total content of PAHs was in range from 62% to 85% in the first stage. Creosote oil was unevenly distributed during the impregnation process and concentration of PAHs is not uniform. Sample-to-solvent ratio does not impact extraction process in the first stage nor could it be observed due to existing inhomogeneity of PAHs distribution in test samples.

Average percentage distribution of the total content of PAHs in the first stage was 76.67%. Average percentage distribution of the total content of PAHs in the second stage (C′) was 20.78% and for the third stage it was 2.33%. Sample-to-solvent ratio does not impact the extraction process neither in the second nor in the third stage (C″). Practically, the entire content of pollutants is isolated in the first three stages. In the first two stages, 97.45% of the total content of pollutants is extracted and with the third stage it is 99.78%. In the fifth stage (C′′′′), there were almost no identified pollutants. During sample preparation of environmental samples for target analysis, extraction stage is almost always performed as one-stage batch. Solid–liquid extractions of WRT test samples with shaking proved that the majority of pollutants was extracted in the first stage, but not the total quantity. Solvent extraction of WRTs has primarily been conducted for monitoring [47], screening [49] or method development [61] for sample preparation prior to analysis. Dichloromethane and *n*-hexane/acetone mixtures have been reported as the most efficient extraction solvents, which is consistent with the results obtained in this study.

In this study, we applied a novel approach to WRT treatment. It is shown through leaching experiments that solvent extraction with organic solvents could be employed as waste treatment for WRT. There was no need for concentration or cleaning up of extracts, because concentration of PAHs in WRT samples is high enough. Standard PAHs profile of creosote extracted from railway ties in dichloromethane is given in Table S3 in the Supplementary Material. As presented in Table S3, the relative ratio of pollutants remains constant through extraction stages. It is not in correlation with the type of solvent, sample-to-solvent ratio or the extraction time. Every solvent extract that is employed in this study shows the same standard PAHs profile. Five individual PAHs classified as potentially carcinogenic to humans, according to IARC, are present in WRT test sample dichloromethane extracts. Their share in relation to other standard PAHs is around 7%. Along with standard PAHs, extracts contain other PAHs and their derivatives, not presented in the standard solution, phenolic compounds and heterocyclic compounds containing nitrogen, sulfur or oxygen that are listed in Table S1 in the Supplementary Material.

Experiments without heating, second and third set, proved that it is possible to extract the vast majority of pollutants from WRT through simple methods, at room temperature with low energy consumption. In the case of solid–liquid multistage batch extraction with shaking, nearly the same amount of pollutants could be isolated in three stages during 45 min, for the same sample-to-solvent ratio as in the case of solid–liquid batch extraction without shaking for 24 h. Soxhlet extraction was applied for the purpose of complete refining of WRT from pollutants. The specified treatment procedure could be designed for large-scale operations, with benefits such as room temperature during extraction, no additional chemical components involved in process, simple phase separation, drying, solvent regeneration by distillation and decontaminated material ready for reuse.

There are other toxic pollutants than PAHs present in the extract that are only qualitatively identified. The proposed extraction process in this study did not include other pollutants which may be present in the WRTs. The results obtained in this study relate only to one investigated WRT and prove the principle of the use of solvent extraction. Since creosote oil-preservation procedures are not regulated by international standards, diverse exposure to weather conditions and different service life of railroad sleepers could result in variable PAH recoveries during extraction. These issues would be the scope of future international R&D on WRT treatment.

## 3. Materials and Methods

### 3.1. Materials

One WRT, obtained from a landfill disposal site in Serbia, was used for all extraction processes in this study. WRT chips with average dimensions of 16 × 0.5 × 0.3 mm (length, width, thickness) were chipped from the used railroad tie with the stainless-steel milling machine (industrial type). Shredding the WRT provides a larger contact surface of the sample with the solvent, accelerates the extraction and increases the efficiency of the extraction. Also, shredding the WRT into pieces of the specified dimensions proved to be very practical for further material handling. The whole waste railroad tie was grinded and well-mixed to obtain a homogenous sample in relation to the matrix and contaminants. Sample tests were prepared from homogenized samples as appropriate portions. As WRT samples have an unpleasant odor because of toxic pollutants, it is desirable to be in minimum contact with them.

Reference material was PAH-Mix 14 (Dr. Ehrenstorfer, LGC, Augsburg, Germany), 2000 μg/mL each component in acetone/benzene. Solvents used for extractions were ethanol 96% (CARLO ERBA Reagents GmbH, Heidelberg, Germany) propan-2-ol (CARLO ERBA Reagents GmbH, Heidelberg, Germany), deionized water, dichloromethane (Fisher chemical, Thermo Fischer Brand, Waltham, MA, USA), acetone (CARLO ERBA Reagents GmbH, Heidelberg, Germany), *n*-hexane (CARLO ERBA Reagents GmbH, Heidelberg, Germany), mixture *n*-hexane/acetone (V/V = 1/1), cyclohexane (CARLO ERBA Reagents GmbH, Heidelberg, Germany), methanol (Fisher chemical, Thermo Fischer Brand, Waltham, MA, USA), N,N-dimethyl formamide (DMF) (Fisher chemical, Thermo Fischer Brand, Waltham, MA, USA), toluene (CARLO ERBA Reagents GmbH, Heidelberg, Germany), ethyl acetate (CARLO ERBA Reagents GmbH, Heidelberg, Germany), acetonitrile (CARLO ERBA Reagents GmbH, Heidelberg, Germany), amyl acetate (CARLO ERBA Reagents GmbH, Heidelberg, Germany), medical gasoline (CARLO ERBA Reagents GmbH, Heidelberg, Germany), *n*-pentane (CARLO ERBA Reagents GmbH, Heidelberg, Germany) and *n*-butyl acetate (CARLO ERBA Reagents GmbH, Heidelberg, Germany), all HPLC grade purity.

Different extraction conditions were tested: type of solvent, sample-to-solvent ratio and extraction time. Extractions were performed at room temperature, 25 °C. Minimum mass appropriate for handling and examination was chosen to be 1 g for soaking tests. WRT chips are voluminous compared with their mass and for 300 cm^3^ Erlenmeyer flasks, 40 cm^3^ was optimum solvent volume for complete immersion of WRT chips.

Three sets of experiments were designed to investigate which one among 15 traditional organic solvents can be used for WRT treatment: Soxhlet extraction, solid–liquid single stage batch extraction (leaching) without shaking, solid–liquid multistage batch extraction (leaching) with shaking.

The dry matter content of creosote-treated wooden railroad tie chips was determined using a gravimetric method. Prior to analysis, the WRT was mechanically reduced into chips and homogenized to obtain representative test samples. Approximately 2 g of two test samples were weighed to determine the initial mass. The samples were dried in a laboratory oven (Binder Model FD-S 115, Tuttlingen, Germany) at 105 ± 2 °C until constant mass was achieved, with intermediate weighing after cooling in a desiccator. Drying and weighing cycles were repeated until the mass difference between two consecutive measurements did not exceed 0.1%. The final mass was recorded as the dry mass. The dry matter content was calculated as the ratio of the dry mass to the initial mass and expressed as a percentage.

### 3.2. Soxhlet Extraction of WRT Chips

One group of experiments represents a well-known Soxhlet extraction technique with glass apparatus performed on wooden chips from creosote-impregnated WRT. Following solvents were employed for this purpose: ethanol 96%, propan-2-ol, deionized water, dichloromethane and acetone. During experiments, the solvent was heated until its boiling point in eight cycles. Test samples from WRT were placed in cellulose extraction thimbles, in the extraction chamber of the apparatus. Four grams of each WRT test sample was weighed and extracted with 150 cm^3^ of solvent. Sample-to-solvent ratio for Soxhlet extraction is driven by the requirements of Soxhlet apparatus, extraction chamber volume, cellulose extraction thimbles dimensions and flask’s volume. Measured values were mass of test sample m_s_ = 4.00 g; solvent volume, V_0_ = 150.0 cm^3^ and extract volume, V_1_. Calculated features were mass of solvent, m_0_; solvent volume retained on test sample, ΔV; mass of solvent retained on test sample, Δm (Δm = ΔV × ρ), solvent mass fraction retained on test sample, Δm/m_0_ and mass of solvent retained on test sample per g, Δm/m_s_.

### 3.3. Solid–Liquid Extraction (Leaching) Through Soaking, Without Shaking

Experiments were conducted using the single-stage batch extraction technique. Extraction time was 24 h at room temperature, 25 °C without shaking, just WRT chips to sink. During extraction time, 1 g of sample was soaked in 40 cm^3^ of solvent. Each probe was put in an Erlenmeyer flask with a neck fitted with ground glass stoppers. The following solvents were employed for this purpose: ethanol 96%, propan-2-ol, deionized water, dichloromethane, acetone, *n*-hexane, mixture *n*-hexane/acetone (V/V = 1/1), cyclohexane, methanol, N,N-dimethyl formamide, toluene, ethyl acetate, acetonitrile, amyl acetate, medical gasoline, *n*-pentane and *n*-butyl acetate. Optimal conditions for solid–liquid extraction of WRT chips through soaking were adjusted to 1 g of test samples, 40 cm^3^ of solvent and 24 h of extraction time at room temperature. Extraction time was set to ensure diffusion and establish an equilibrium state in the redistribution of pollutants. Diffusion is a relatively slow process and is more pronounced during extraction with solvents with less extraction capacity, such as cyclohexane, toluene and *n*-hexane. Measured features were mass of test sample m_s_ = 1.00 g; solvent volume, V_0_ = 40.0 cm^3^; extract volume, V_1_. Calculated features were mass of solvent, m0; solvent volume retained on test sample, ΔV; mass of solvent retained on test sample, Δm (Δm = ΔV × ρ), solvent mass fraction retained on test sample, Δm/m_0_ and mass of solvent retained on test sample per g, Δm/m_s_.

### 3.4. Solid–Liquid Extraction (Leaching) with Shaking

Dichloromethane as extraction solvent was chosen for the third set of investigations, based on multistage batch solvent extraction technique with addition of fresh solvent to filtration cake in each stage, after decanting extract from the previous step. Cellulose paper (Whatman^®^ Grade 1573 FF, Cytiva, Marlborough, MA, USA) is used for decanting and gravity filtration. An aliquot of 1.00 cm^3^ after the filtration step was taken for GC-FID analysis of 16 US EPA PAHs. In the third set of experiments, solid–liquid extraction with shaking, solid-to-liquid ratio was set to be higher and smaller than in the solid–liquid extraction through soaking, without shaking. Investigation was performed to determine how many batch extraction stages are needed to extract pollutants until the limit of detection. Extraction time of railroad tie test samples is reduced to 2.00 h, 1.00 h and 0.25 h. To meet practical requirements and needs, 0.25 h left enough time for decanting and filtering of extracts and for preparation of the next batch stage. The extraction scheme includes test samples of 5.00 g in 300 cm^3^ Erlenmeyer flasks, with distinct solvent volume (100.0 cm^3^, 200.0 cm^3^ and 300.0 cm^3^) and varied extraction time (0.25 h, 1.00 h and 2.00 h). An aliquot of 1.00 cm^3^ after gravity filtration was taken for GC-FID quantitative analysis of 16 U.S. EPA PAHs. Successive extractions were achieved till the sum of 16 U. S. EPA PAHs was below the limit of detection.

### 3.5. Quantification of 16 U.S. EPA PAHs and Identification of Organic Pollutants

Liquid samples were prepared by filtering aliquots of extract through membrane nylon filters (Whatman Uniflo^®^, Cytiva, Marlborough, MA, USA) without the cleaning-up procedure. Gas chromatography separation and quantification was conducted on Gas Chromatograph Nexis GC-2030, with LabSolutions software, Version 5.97, Shimadzu Corporation (Nakagyo-ku, Kyoto, Japan), equipped with Autosampler AOC-20i, split/splitless injection unit (SPL), capillary column Thermo TG-4MS, length 30 m × 0.25 mm I.D, 0.25 µm-thick film (Thermo Fisher Scientific, Waltham, MA, USA). The GC temperature program of the method for the detection of PAHs is as follows: the initial temperature of the column is 40 °C and the hold time is 4 min. Then, the temperature increases at a rate of 10 °C/min until it reaches 300 °C. The hold time at this temperature is 15 min. The entire run lasts 45 min. Detection of analytes was performed with Flame Ionization Detector (FID) at temperature 315 °C. Qualitative analysis and identification of organic pollutants was performed by GC-MS technique on Gas Chromatograph Agilent Technologies 7890B GC System with mass detector Agilent Technologies 5977MSD and NIST compounds library (Agilent MassHunter Workstation Software, Version 10.1) was employed to identify organic polluting substances (Santa Clara, CA, USA). External calibration was used and calibration plots were achieved using five different concentration levels of the 16 PAHs. Satisfactory coefficients of determination (0.984–0.999) were obtained for all the 16 PAHs in the range of 0.5–20.0 μg/mL. The minimum concentration of analyte that can be identified by the method is referred to as the limit of detection (LOD). Measurements were performed in duplicate (n = 2). The total PAH content was determined independently for two test samples and the standard deviation was calculated as standard deviation based on a sample. All results are expressed as percentages with associated standard deviations (mean ± SD). The standard deviation of summed values was calculated assuming independent measurements. The standard deviation of the mean was obtained using the combined standard deviation, considering individual measurement uncertainties.

## 4. Conclusions

The aim of this work was to reduce the concentration of pollutants in used creosote-impregnated wooden railroad ties that have become waste. According to the objective, the following three sets of experiments were designed: Soxhlet extraction, solid–liquid single stage batch extraction (leaching) without shaking and solid–liquid multistage cross-current batch extraction with shaking. Except deionized water, the rest of four Soxhlet extracts represent recovery of PAHs over 7%, in a series dichloromethane > ethanol 96% > acetone > propan-2-ol. Measured Soxhlet extracted mass share in different solvents suggests each among them could be employed for the extraction process on a large scale.

Extraction capacity of solvents solid–liquid single stage batch extraction without shaking decreases in a series dichloromethane > *n*-hexane/acetone > acetone > methanol > ethanol 96% > propan-2-ol > cyclohexane > toluene > *n*-hexane. Aforementioned solvents could be employed in order to accomplish leaching treatment of WRT chips at room temperature with the decrease in pollutants concentration. The highest measured value 7.89% in both sets of experiments corresponds to dichloromethane Soxhlet extract. The highest measured value in solid–liquid extraction without shaking tests was 7.50% and indicates that dichloromethane maintains the best extraction capacity among tested solvents. The lowest extracted mass share 1.94%, except deionized water, was measured in DMF extract. The rest of tested solvents, ethyl acetate, acetonitrile, amyl acetate, medical gasoline, *n*-pentane and *n*-butyl acetate, have measured extracted mass share below 3.50%, which is less than a half of the highest measured values. Deionized water came out with expected low values for PAHs extraction, due to high hydrophobicity of PAHs. Previously mentioned solvents should not be an option for treatment of WRT chips in order to remove sixteen priority PAHs.

Solid–liquid multistage batch extraction tests with shaking pointed that sample-to-solvent ratio does not impact extraction process with dichloromethane, or it could not be observed due to existing inhomogeneity of PAHs distribution in the WRT test samples. The average total content of PAHs was 7.62% for various extraction times. Average recovery of the total content of PAHs in the first stage was 76.67%, in the second stage was 20.78% and for the third stage it was 2.33%. Practically the entire content of pollutants is isolated in the first three stages. In the first two stages 97.45% of total content of pollutants is extracted. Four stages during 1 h in the batch solvent extraction process with shaking are sufficient to perform reduction in the sixteen priority PAHs from WTR chips below the limit of detection. The specified treatment procedure could be upgraded for large-scale operations, with benefits such as room temperature during extraction, no additional chemical components involved in the process, simple phase separation, drying step and solvent regeneration by distillation.

## Figures and Tables

**Table 1:** Solvent fraction retained on test sample, Δm/m_0_ and mass of solvent retained on test sample per g, Δm/m_s_ by Soxhlet extraction.

Solvent	Solvent Density, ρ (g/cm^3^)	Mass of Solvent, m_0_ (g)	Extract Volume, V_1_ (cm^3^)	Solvent Volume Retained on Test Sample, ΔV (cm^3^)	Mass of Solvent Retained on Test Sample, Δm (g)	Solvent Fraction Retained on Test Sample, Δm/m_0_	Mass of Solvent Retained on Test Sample per g, Δm/m_s_
Ethanol 96%	0.789	118.35	80.0	70.0	55.23	0.47	13.81
Propan-2-ol	0.780	117.00	84.0	66.0	51.48	0.44	12.87
Water deionized	0.997	149.55	88.0	62.0	61.81	0.41	15.45
Dichloromethane	1.325	198.75	69.0	81.0	107.32	0.54	26.83
Acetone	0.784	117.60	84.0	66.0	51.74	0.44	12.93

**Table 2:** Solvent mass fraction retained on test sample by solid–liquid extraction through soaking, without shaking, Δm/m_0_ and mass of solvent retained on test sample per g, Δm/m_s_.

Solvent	Solvent Density, ρ (g/cm^3^)	Mass of Solvent, m_0_ (g)	Extract Volume, V_1_ (cm^3^)	Solvent Volume Retained on Test Sample, ΔV (cm^3^)	Mass of Solvent Retained on Test Sample, Δm (g)	Solvent Mass Fraction Retained on Test Sample, Δm/m_0_	Mass of Solvent Retained on Test Sample per g, Δm/m_s_
Ethanol 96%	0.789	31.56	34.5	5.5	4.34	0.14	4.34
Propan-2-ol	0.780	31.2	35.0	5.0	3.90	0.12	3.90
Water deionized	0.997	39.88	32.5	7.5	7.48	0.18	7.48
Dichloromethane	1.325	53.00	33.0	7.0	9.27	0.17	9.27
Acetone	0.784	31.36	34.0	6.0	4.70	0.15	4.70
*n*-Hexane	0.655	26.2	35.0	5.0	3.27	0.12	3.27
*n*-Hexane/acetone *	0.706	28.24	34.0	6.0	4.24	0.15	4.24
Cyclohexane	0.774	30.96	34.5	5.5	4.84	0.15	4.84
Methanol	0.792	31.68	34.5	5.5	4.29	0.14	4.29
DMF	0.948	37.92	34.0	6.0	5.64	0.15	5.64
Toluene	0.862	34.48	35.0	5.0	4.35	0.12	4.35
Ethyl acetate	0.902	36.8	34.0	6.0	5.41	0.15	5.41
Acetonitrile	0.786	31.44	33.0	7.0	5.50	0.17	5.50
Amyl acetate	0.876	35.04	36.0	4.0	3.50	0.10	3.50
Medical gasoline	0.780	31.2	33.0	7.0	5.46	0.17	5.46
*n*-Pentane	0.626	25.04	24.0	16.0	10.16	0.40	10.16
*n*-Butyl acetate	0.882	35.28	36.0	4.0	3.53	0.10	3.53

* (V/V = 1/1).

**Table 3:** Extracted mass fraction of the 16 U.S. EPA PAHs in test samples using dichloromethane (solid–liquid extraction, shaking), presented as mean ± SD (n = 2).

Extraction Time (h)		2.00			1.00			0.25		
Solvent Volume, V_0_ (cm^3^)		100.0	200.0	300.0	100.0	200.0	300.0	100.0	200.0	300.0
Series		C1	C2	C3	C4	C5	C6	C7	C8	C9
Mass share of PAHs in test sample (%)	Stage 1	5.37 ± 0.42	6.14 ± 0.34	6.52 ± 0.25	5.04 ± 0.40	5.81 ± 0.33	6.23 ± 0.25	5.17 ± 0.42	5.40 ± 0.34	6.45 ± 0.27
	Stage 2	1.63 ± 0.15	1.83 ± 0.13	0.82 ± 0.10	1.35 ± 0.13	1.11 ± 0.12	3.12 ± 0.09	2.91 ± 0.15	0.79 ± 0.13	1.11 ± 0.07
	Stage 3	0.23 ± 0.07	0.17 ± 0.06	0.13 ± 0.06	0.25 ± 0.06	0.16 ± 0.05	0.08 ± 0.05	0.28 ± 0.08	0.12 ± 0.06	0.19 ± 0.07
	Stage 4	0.03 ± 0.01	0.01 ± 0.01	<LOD	0.02 ± 0.01	0.01 ± 0.01	0.04 ± 0.01	0.03 ± 0.01	0.04 ± 0.01	0.01 ± 0.01
	Stage 5	<LOD	<LOD	<LOD	<LOD	<LOD	<LOD	0.010 ± 0.01	0.010 ± 0.01	<LOD
	Sum of extracted mass shares (%)	7.26 ± 0.45	8.15 ± 0.37	7.47 ± 0.28	6.66 ± 0.43	7.09 ± 0.36	9.47 ± 0.27	8.40 ± 0.45	6.36 ± 0.37	7.76 ± 0.29

Limit of detection (LOD = 0.002%).

## Data Availability

The original contributions presented in the study are included in the article, further inquiries can be directed to the corresponding authors.

## Supplementary Materials

The following supporting information can be downloaded at https://www.mdpi.com/article/10.3390/molecules31030406/s1. Table S1. Qualitative analysis of WRT test sample dichloromethane extract by GC-MS. Table S2. Recovery of 16 US EPA PAHs in test sample. Table S3. Standard PAHs profile (%) of creosote extracted from railway ties in dichloromethane.
