# Supplementary material for: Utilization of Organic Solvents for the Recycling of Waste Wooden Railroad Ties
Source: Molecules. 2026 Jan 24;31(3):406. doi: 10.3390/molecules31030406 (PMC12899867; doi:10.3390/molecules31030406)
Supplement: Supplementary file 1 [file molecules-31-00406-s001.zip › molecules-4070282-supplementary.pdf]

Supplementary material:

Table S1. Qualitative analysis of WRT test sample dichloromethane extract by GC-MS.

Serial number	Compound	Retention time, R_t (min)	Molecular formula
1	Phenol	9.45	C_6H_6O
2	Benzyl alcohol	10.78	C_7H_8O
3	3-methyl phenol	11.13	C_7H_8O
4	2,6-dimethyl phenol	12.33	$C_8H_{10}O$
5	2,4-dimethyl phenol	12.64	$C_8H_{10}O$
6	Naphthalene* (Nap)	12.96	$C_{10}H_8$
7	Quinoline	13.77	C_9H_7N
8	Isoquinoline	14.09	C_9H_7N
9	1-methyl naphthalene	14.61	$C_{11}H_{10}$
10	2-methyl quinoline	14.79	$C_{10}H_9N$
11	2-methyl naphthalene	14.86	$C_{11}H_{10}$
12	2-methyl isoquinoline	15.40	$C_{10}H_9N$
13	Biphenyl	15.77	$C_{12}H_{10}$
14	2-(2-hydroxyethyl)naphthalene	15.98	$C_{12}H_{12}O$
15	1-isopropyl naphthalene	16.12	$C_{13}H_{14}$
16	Acenaphthene* (Ace)	17.22	$C_{12}H_{10}$
17	1-isocyano naphthalene	17.31	$C_{11}H_7N$
18	Dibenzofuran	17.61	$C_{12}H_8O$
19	Fluorene* (Fle)	18.43	$C_{13}H_{10}$
20	1-allylnaphthalene	18.55	$C_{13}H_{12}$
21	2-aminofluorene	18.84	$C_{13}H_{11}N$
22	1-hydroxyacenaphthene	19.33	$C_{12}H_{10}O$
23	Fluorenone	20.20	$C_{13}H_8O$
24	1,1'-biphenyl-4,4'-diamine (benzidine)	20.37	$C_{12}H_{12}N_2$
25	Phenanthrene* (Ph)	20.74	$C_{14}H_{10}$
26	Anthracene* (An)	20.81	$C_{14}H_{10}$
27	Carbazole	21.22	$C_{12}H_9N$
28	9-(chloromethyl)anthracene	21.85	$C_{15}H_{11}Cl$
29	Fluoranthene* (Fla)	23.56	$C_{16}H_{10}$
30	Pyrene* (Py)	24.06	$C_{16}H_{10}$
31	Benz(a)anthracene* (B[a]A)	26.93	$C_{18}H_{12}$
31	Crysene* (Chr)	27.01	$C_{18}H_{12}$
32	Benzo(b)fluoranthene* (B[b]F) and Benzo(k)fluoranthene* (B[k]F)	29.36	$C_{20}H_{12}$
33	Benzo(a)pyrene* (B[a]P)	30.01	$C_{20}H_{12}$

*PAHs from the Reference material

Table S2. Recovery of 16 US EPA PAHs in test sample.

Solvent	Leaching through soaking Extracted mass share of 16 U.S. EPA PAHs in test samples in V_0, %	Soxhlet extraction, heating Extracted mass share of 16 U.S. EPA PAHs in test samples in V_1, %
1. Ethanol 96%	6.11	7.74
2. Propan-2-ol	5.62	7.24
3. Water deionized	<0.002[1]	<0.002[1]
4. Dichloromethane	7.50	7.89
5. Acetone	6.82	7.26
6. *n*-Hexane	5.22	-
7. *n*-Hexane/acetone*	7.45	-
8. Cyclohexane	5.56	-
9. Methanol	6.78	-
10. DMF	1.94	-
11. Toluene	5.45	-
12. Ethyl acetate	3.31	-
13. Acetonitrile	2.99	-
14. Amyl acetate	2.02	-
15. Medical gasoline	2.58	-
16. *n*-Pentane	3.19	-
17. *n*-Butyl acetate	2.55	-

[1]below limit of quantification, *(V/V=1/1)

Table S3. Standard PAHs profile (%) of creosote extracted from railway ties in dichloromethane.

Series	C1, %	C2, %	C3, %	C4, %	C5, %	C6, %	C7, %	C8, %	C9, %
Naphtalene	1	1	1	0	1	1	1	0	1
Acenaphthylene	0	0	0	0	0	0	0	0	0
Acenaphtene	11	11	12	11	12	12	11	11	11
Fluorene	12	12	12	13	13	12	13	12	12
Phenenthrene	31	32	31	33	33	33	35	33	33
Anthracene	8	8	9	9	9	9	8	9	10
Fluoranthene	16	16	15	16	15	15	15	15	14
Pyrene	12	11	11	10	9	11	9	11	10
Benz[a]anthracene	3	3	3	3	3	3	3	3	3
Chrysene	4	4	4	4	3	3	3	4	4
Benzo[b]fluoranthene	1	1	1	1	1	1	1	1	1
Benzo[k]fluoranthene	0	0	0	0	0	0	0	0	0
Benzo[a]pyrene	1	1	1	0	1	0	1	1	1
Dibenz[a,h]anthracene	0	0	0	0	0	0	0	0	0
Benzo[g,h,i]perylene	0	0	0	0	0	0	0	0	0
Indeno[1,2,3-c,d]pyrene	0	0	0	0	0	0	0	0	0
Mass share of 16 US EPA PAHs in test sample	7.3	8.2	7.5	6.7	7.1	9.5	8.4	6.3	7.7
